# Use of a Cytosponge biomarker panel to prioritise endoscopic Barrett's oesophagus surveillance: a cross-sectional study followed by a real-world prospective pilot

**DOI:** 10.1016/S1470-2045(21)00667-7

**Published:** 2022-02

**Authors:** Nastazja Dagny Pilonis, Sarah Killcoyne, W Keith Tan, Maria O'Donovan, Shalini Malhotra, Monika Tripathi, Ahmad Miremadi, Irene Debiram-Beecham, Tara Evans, Rosemary Phillips, Danielle L Morris, Craig Vickery, Jon Harrison, Massimiliano di Pietro, Jacobo Ortiz-Fernandez-Sordo, Rehan Haidry, Abigail Kerridge, Peter D Sasieni, Rebecca C Fitzgerald

**Affiliations:** a MRC Cancer Unit, Hutchison-MRC Research Centre, University of Cambridge, Cambridge, UK; b Department of Histopathology, Addenbrooke's Hospital, Cambridge, UK; c Department of Gastroenterology, Princess Alexandra Hospital NHS Trust, Harlow, UK; d Department of Gastroenterology, East and North Herts NHS Trust, Stevenage, UK; e Department of Surgery, West Suffolk Hospital, Bury St Edmunds, UK; f Department of Gastroenterology, Harrogate District Hospital, Harrogate, UK; g Nottingham Digestive Diseases Centre and NIHR Nottingham Biomedical Research Centre, Nottingham University Hospitals NHS Trust and University of Nottingham, Nottingham, UK; h Cancer Prevention Group in Clinical Trials Unit, King's Clinical Trials Unit, King's College London, London, UK

## Abstract

**Background:**

Endoscopic surveillance is recommended for patients with Barrett's oesophagus because, although the progression risk is low, endoscopic intervention is highly effective for high-grade dysplasia and cancer. However, repeated endoscopy has associated harms and access has been limited during the COVID-19 pandemic. We aimed to evaluate the role of a non-endoscopic device (Cytosponge) coupled with laboratory biomarkers and clinical factors to prioritise endoscopy for Barrett's oesophagus.

**Methods:**

We first conducted a retrospective, multicentre, cross-sectional study in patients older than 18 years who were having endoscopic surveillance for Barrett's oesophagus (with intestinal metaplasia confirmed by TFF3 and a minimum Barrett's segment length of 1 cm [circumferential or tongues by the Prague C and M criteria]). All patients had received the Cytosponge and confirmatory endoscopy during the BEST2 (ISRCTN12730505) and BEST3 (ISRCTN68382401) clinical trials, from July 7, 2011, to April 1, 2019 (UK Clinical Research Network Study Portfolio 9461). Participants were divided into training (n=557) and validation (n=334) cohorts to identify optimal risk groups. The biomarkers evaluated were overexpression of p53, cellular atypia, and 17 clinical demographic variables. Endoscopic biopsy diagnosis of high-grade dysplasia or cancer was the primary endpoint. Clinical feasibility of a decision tree for Cytosponge triage was evaluated in a real-world prospective cohort from Aug 27, 2020 (DELTA; ISRCTN91655550; n=223), in response to COVID-19 and the need to provide an alternative to endoscopic surveillance.

**Findings:**

The prevalence of high-grade dysplasia or cancer determined by the current gold standard of endoscopic biopsy was 17% (92 of 557 patients) in the training cohort and 10% (35 of 344) in the validation cohort. From the new biomarker analysis, three risk groups were identified: high risk, defined as atypia or p53 overexpression or both on Cytosponge; moderate risk, defined by the presence of a clinical risk factor (age, sex, and segment length); and low risk, defined as Cytosponge-negative and no clinical risk factors. The risk of high-grade dysplasia or intramucosal cancer in the high-risk group was 52% (68 of 132 patients) in the training cohort and 41% (31 of 75) in the validation cohort, compared with 2% (five of 210) and 1% (two of 185) in the low-risk group, respectively. In the real-world setting, Cytosponge results prospectively identified 39 (17%) of 223 patients as high risk (atypia or p53 overexpression, or both) requiring endoscopy, among whom the positive predictive value was 31% (12 of 39 patients) for high-grade dysplasia or intramucosal cancer and 44% (17 of 39) for any grade of dysplasia.

**Interpretation:**

Cytosponge atypia, p53 overexpression, and clinical risk factors (age, sex, and segment length) could be used to prioritise patients for endoscopy. Further investigation could validate their use in clinical practice and lead to a substantial reduction in endoscopy procedures compared with current surveillance pathways.

**Funding:**

Medical Research Council, Cancer Research UK, Innovate UK.

## Introduction

Oesophageal adenocarcinoma is a public health concern due to increasing incidence rates; as it generally presents at an advanced stage, there is a poor 5-year overall survival of less than 20%, with little improvement over the past 30 years.^1^, ^2^ Barrett's oesophagus is a precancerous lesion that increases the risk of oesophageal adenocarcinoma through intermediary stages of increasing morphological abnormality on histology, described as non-dysplastic Barrett's oesophagus, low-grade dysplasia, high-grade dysplasia, and invasive adenocarcinoma.^3^ Given the poor prognosis of advanced oesophageal adenocarcinoma, societal guidelines recommend that patients diagnosed with non-dysplastic Barrett's oesophagus should be enrolled into regular endoscopic surveillance to allow detection of dysplasia, which can be treated effectively endoscopically.^4^, ^5^, ^6^, ^7^, ^8^ However, with an estimated progression rate for non-dysplastic Barrett's oesophagus of 0·3% per year, most patients will not progress.^9^, ^10^ Identification of individuals at high risk of Barrett's oesophagus neoplasia could allow us to prioritise endoscopy resources for those who will benefit most from endoscopic surveillance, thus decreasing the burden of endoscopy for patients and health-care systems.^11^, ^12^, ^13^ This prioritisation of resources has been of particular concern during the COVID-19 pandemic, when endoscopic surveillance has been limited due to concerns around infection risks from the aerosol-generating nature of this procedure and re-allocation of resources.^14^

**Research in context**

**Evidence before this study**

We searched PubMed from March 1, 2019, to July 31, 2019, for publications in English on non-endoscopic surveillance in Barrett's oesophagus, using the terms “Barrett's (o)esophagus” AND “surveillance” AND “non-endoscopic”. This search retrieved nine articles, seven of which were reviews. Only two articles related to new methods of non-endoscopic surveillance. One study utilised electronic health records to identify patients on the basis of known clinical demographic risks, and the other was our initial publication that used the non-endoscopic oesophageal cell collection device known as the Cytosponge alongside clinical demographic and biomarker information in a prospective cohort study. As earlier detection vastly improves outcomes in oesophageal adenocarcinoma, patients with the precursor lesion Barrett's oesophagus are recommended to undergo regular endoscopic surveillance to detect and treat early neoplasia. However, regular endoscopy is burdensome for patients and health services while most patients with Barrett's oesophagus will not develop cancer, and the COVID-19 pandemic has exacerbated endoscopy bottlenecks.

**Added value of this study**

We developed and tested a simple Cytosponge biomarker panel for use in surveillance of patients with known Barrett's oesophagus. Specifically, cytological assessment of cellular atypia and p53 overexpression with immunohistochemistry could be used to identify patients who are likely to have high-grade dysplasia and early cancer. Use of clinical risk factors enables one to distinguish between low-risk and moderate-risk individuals who have a negative Cytosponge result. During the COVID-19 pandemic, we have shown that this triage approach can efficiently prioritise patients for endoscopy, by contrast with the current pathway in which all patients have regular endoscopy even though most patients with Barrett's oesophagus will not progress.

**Implications of all the available evidence**

Implementation of Cytosponge in Barrett's oesophagus surveillance programmes could enable prioritisation of patients for endoscopic confirmation and therapy. Furthermore, patients deemed to be at low risk of progression (no clinical risk factors or Cytosponge findings, which accounts for >50% of patients) could potentially avoid endoscopy and have Cytosponge follow-up. Especially in view of COVID-19-related delays, and concerns regarding the greater potential of transmission of infection during endoscopy, this could have substantial benefits for patients and the health-care system. Further prospective studies will aim to validate these findings, determine the optimal frequency of Cytosponge, and to quantify the economic and psychological impact of reduced endoscopy procedures.

A non-endoscopic oesophageal cell collection device known as the Cytosponge, coupled with a biomarker test for trefoil factor 3 (TFF3), has been shown to increase detection of Barrett's oesophagus ten times over the current standard of care.^15^ As a logical extension of this finding, additional biomarkers could be applied to Cytosponge to identify dysplasia and carcinoma. We have previously shown that a panel encompassing p53 immunohistochemistry and *TP53* mutation, aurora kinase A (AUKA; a surrogate for aneuploidy), and clinical factors (age and length of Barrett's oesophagus) was able to predict patients with high-grade dysplasia or intramucosal cancer with good accuracy (area under the receiver operating characteristic curve [AUROC] 0·87; 99% CI 0·73–0·95).^16^

The clinical applicability of this Cytosponge risk stratification panel is limited because it requires *TP53* gene sequencing from formalin-fixed, paraffin-embedded samples, and the AUKA immunohistochemistry is complex to score and the antibody is no longer commercially available.^16^ As the majority of the risk from the previous logistic regression analysis derived from the p53 immunohistochemistry and haematoxylin and eosin stain for cellular atypia, we hypothesised that these biomarkers would effectively identify patients with high-grade dysplasia or intramucosal cancer.^17^ We also reconsidered the relevance of clinical risk factors, as the objective is to make any algorithm as sensitive as possible and ensure its clinical applicability.

In this study, we aimed to derive and evaluate Cytosponge biomarkers and clinical risk factors to triage patients at high risk, moderate risk, and low risk of Barrett's oesophagus-related neoplasia. We determined the most informative biomarkers to derive a clinically applicable decision tree to classify the risk for prevalent dysplasia. As a practical demonstration of this approach, we performed a prospective study in the context of restricted endoscopy services during the COVID-19 pandemic.

## Methods

### Study design and participants

We first conducted a retrospective, multicentre, cross-sectional study in patients with known Barrett's oesophagus who had a Cytosponge test followed by endoscopy.

We included all available consecutive patients older than 18 years with a confirmed diagnosis of Barrett's oesophagus (with intestinal metaplasia confirmed by TFF3 and a minimum Barrett's segment length of 1 cm [tongues or circumferential by the Prague C and M criteria]) who were having endoscopic surveillance as part of the BEST2 (ISRCTN12730505) and BEST3 (ISRCTN68382401) clinical trials.^15^, ^16^ All patients in these trials received Cytosponge and confirmatory endoscopy from July 7, 2011, to April 1, 2019. Risk stratification was a stated secondary aim of these trials. Patients were recruited from across hospitals that were geographically dispersed across England. Eligible participants were split into training (n=557) and validation (n=334) cohorts on the basis of date of recruitment (training cohort 2011–13, validation cohort 2013 onwards).

Ethics approval was obtained from the East of England—Cambridge Central Research Ethics Committee (BEST2 10/H0308/71; BEST3 16/EE/0546) and the trials are registered in the UK Clinical Research Network Study Portfolio (9461). Written informed consent was obtained from each patient for participation in the trials.

For the prospective cohort, in response to COVID-19 and the need to provide an alternative to endoscopic surveillance, we developed a protocol within the DELTA implementation study (integrated diagnostic solution for early detection of oesophageal cancer study; funded by Innovate UK; ISRCTN91655550). Ethics approval was obtained from the East of England—Cambridge Central Research Ethics Committee (DELTA 20/EE/0141) and written informed consent was obtained from each patient. The prospective pilot included one tertiary referral centre (Addenbrooke's Hospital, Cambridge), and four community hospitals (Lister Hospital, Stevenage; Princess Alexandra Hospital, Harlow; West Suffolk Hospital, Bury St Edmunds; Harrogate District Hospital, Harrogate) in England. Patients who had their Barrett's oesophagus surveillance delayed due to decreased endoscopy provision during the COVID-19 pandemic were invited to take part in the prospective pilot from Aug 27, 2020. Endoscopy results were available for patients categorised as high risk by Cytosponge.

### Procedures

In the BEST2 and BEST3 trials, patient exposure and demographic information was collected during a single office visit (eg, alcohol, tobacco, drug use). In the DELTA study, a small amount of demographic information was collected at the time of the Cytosponge test. Cytosponge (Europlaz, Southminster, UK for BEST2 and BEST3; Medtronic, Minneapolis, MN, USA for DELTA) was administered by a research nurse in BEST2 and BEST3, and by clinical nurses in DELTA.

After retrieval, the Cytosponge was placed in SurePath Preservative Fluid (TriPath Imaging, Burlington, NC, USA) and kept at 4°C. Both atypia and p53 immunohistochemistry were performed on formalin-fixed, paraffin-embedded slides from the Cytosponge (appendix p 1). Atypia was assessed on the haematoxylin and eosin slide and included both clear dysplasia and atypia of unknown significance. A p53 immunohistochemistry (Novocastra NCL-L-p53-D07, Newcastle, UK; 1:50 dilution, on the BondMax autostainer with the Leica Bond Polymer Detection kit; Leica Biosystems, Milton Keynes, UK) staining with an intensity of 3 was considered significant, as previously published.^18^ The p53 absent staining pattern cannot be reliably ascertained from Cytosponge samples, so these cases could be missed.^16^ Both biomarker tests were considered positive if there was a consensus diagnosis from at least two expert pathologists (MO'D, SM, MT, and AM), and in some cases evaluation of the p53 alongside the haematoxylin and eosin could help to clarify the atypia diagnosis.

Endoscopies were performed by the local gastroenterologist on the same day as Cytosponge (BEST2) or within 2 months of Cytosponge (BEST3). Additional information about the study procedures and definitions are provided in the appendix (pp 1–2).

### Outcomes

The primary outcome was a diagnosis of high-grade dysplasia or cancer detected in any of the endoscopic biopsies. For invasive cancer cases, the degree of invasion was determined from the endoscopic mucosal resection and intramucosal cancer was used to denote cases confined to the mucosal layer (T1a) at the procedure following the Cytosponge test. The objective was to identify a high-risk group for the primary outcomes using any combination of Cytosponge and clinical variables.

The secondary outcome was a diagnosis of any grade of dysplasia (low-grade dysplasia, high-grade dysplasia, or cancer) detected in the corresponding endoscopic biopsies.^19^ Indefinite for dysplasia was not considered abnormal due to the subjectivity of the assessment and poor intra-observer agreement.^20^

### Statistical analysis

All available patients in the retrospective training cohort (n=557) were analysed to evaluate a logistic regression model to assess and select significant risk factors for both primary (high-grade dysplasia or cancer) and secondary (any grade of dysplasia or cancer) endpoints. Aside from the Cytosponge biomarkers, clinical demographic variables from the BEST2 and BEST3 retrospective cohort included continuous variables for age at the time of endoscopy, body-mass index, waist-to-hip ratio, years of surveillance, and maximum length and circumferential length of the Barrett's oesophagus segment (measured in cm as per the Prague classification); and categorical variables for smoking status, sex, previous oesophagitis, years with heartburn, units of alcohol per week, frequency of alcohol use, hiatus hernia, family history of oesophageal cancer, and ethnicity.^15^, ^16^ As previous analyses reported significant differences in age and Barrett's segment length in patients with and without dysplasia, we evaluated those in the retrospective training cohort using a Mann-Whitney *U* test.

Additional methods, such as random forest and generalised boosted regression, were tested to investigate the possibility of developing a robust prediction from weak associations. However, as these provided no additional improvement over standard regression models, these results were not included here.

Each covariate was tested for significance (defined as p≤0·05) in a univariate logistic regression model for the primary and secondary outcomes. Significant covariates were considered in a multivariate logistic regression model. The predictive accuracy of the selected covariates was further assessed using the AUROC to compare the accuracy of the Cytosponge biomarker panel (atypia and p53 overexpression immunohistochemistry) with a model including the Cytosponge biomarker panel or significant clinical demographic variables, or both. 95% CIs with Bootstrap were computed for each AUROC statistic; where clinical features are included, the Youden statistic was used to select the best threshold for sensitivity and specificity. All statistical analyses were done using R version 3.6.2.

Biomarkers (atypia and p53 overexpression immunohistochemistry) and significant clinical demographic variables identified in the logistic regression models were used to generate a simple decision tree using R version 3.6.2. The highest risk group was comprised of biomarker-positive cases, as these identified the greatest proportion of both primary and secondary outcomes in patients. The moderate-risk group was selected to minimise false-negative results. For those patients with no atypia or p53 overexpression on their Cytosponge, clinical risk factors associated with primary and secondary outcomes were used to identify patients at risk, and cutoffs were derived to maximise sensitivity at the expense of specificity. These factors were an ultra-long Barrett's oesophagus segment (maximum length >10 cm or circumferential length >6 cm) or patients with a long segment (maximum length >5 cm or circumferential length ≥3 cm) and either male sex or age older than 60 years. Sensitivity for identification of dysplasia (either primary or secondary outcomes) was evaluated using AUROC for all high-risk and moderate-risk patients versus low-risk patients.

Following analysis in the retrospective cohorts, we evaluated the feasibility of using the decision tree approach in clinical practice within the prospective cohort. The prospective pilot was designed during the COVID-19 pandemic when many patients with Barrett's oesophagus had reduced access to standard surveillance endoscopies, and therefore it was ethically appropriate to use the Cytosponge as a means to triage patients for endoscopy according to their risk of dysplasia or cancer. The endoscopy data for the prospective cohort are still being collected for low-risk and moderate-risk groups and will be published later.

### Role of the funding source

The funders of the study had no role in study design, data collection, data analysis, data interpretation, or writing of the report.

## Results

The retrospective cohorts included a total of 891 patients (557 in the training cohort and 334 in the validation cohort, enrolled consecutively). The cohorts were well matched for baseline characteristics and the profile was as expected for this disease, predominantly male sex (453 [81%] of 557 patients in the training cohort, 250 [75%] of 334 in the validation cohort) with a median age of 65 years (IQR 59–72) and 67 years (58–73), respectively (table 1). The Barrett's oesophagus maximum length (Prague M value) was a median of 5 cm (IQR 3–8) in the training cohort and 3 cm (2–6) in the validation cohort.

**Table 1:** Demographics and endoscopy results of patients included in the retrospective training and validation cohorts and the prospective validation cohort

		**Training cohort (n=557)**	Validation cohort (n=334)*	**Prospective validation cohort (n=223)**
Age, years		65 (59–72)	67 (58–73)	69 (60–74)
Sex				
	Male	453 (81%)	250 (75%)	165 (74%)
	Female	104 (19%)	84 (25%)	58 (26%)
Ethnicity				
	White	545 (98%)	..	..
	Other	11 (2%)	..	..
	Missing	1 (<1%)	..	..
Barrett's oesophagus maximum segment length, cm		5 (3–8)	3 (2–6)	3 (2–6)†
Barrett's oesophagus circumferential length, cm		3 (1–6)	1 (0–4)	1 (0–4)†
Body-mass index, kg/m^2^		28·25 (25·61–31·07)‡	27·90 (25·20–30·81)	26·90 (24·12–29·30)§
Smoking status				
	Never smoked	211 (38%)	128 (38%)	75 (34%)
	Any previous smoking	346 (62%)	192 (58%)	99 (44%)
	Missing	0	14 (4%)	49 (22%)

Data are n (%) or median (IQR). Ethnicity data were not collected for the retrospective validation cohort or the prospective validation cohort.

* 344 samples.

† Data missing for 44 patients.

‡ Data missing for six patients.

§ Data missing for 156 patients.

**Figure 1:**
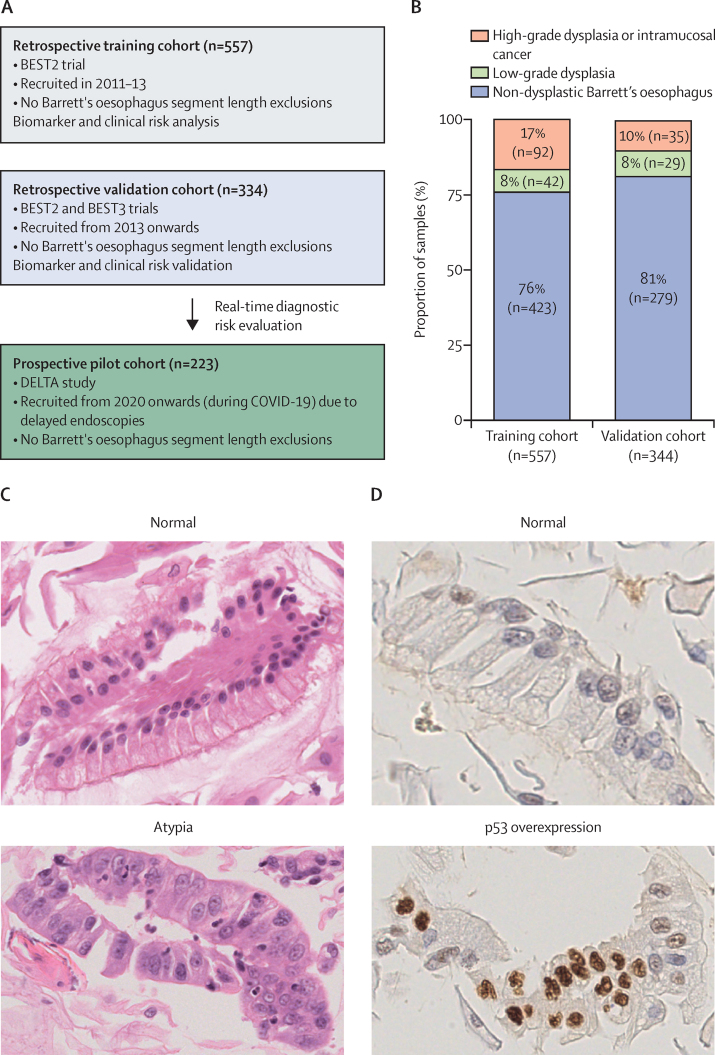
Study design and cohort summary (A) Study design; all patients with Barrett's oesophagus in the BEST2 and BEST3 trials were included in the retrospective training and validation cohorts, and all patient who had been included to date in the DELTA study were included in the prospective pilot cohort. (B) Baseline summary of endoscopic biopsy diagnoses among individuals in the training and validation cohorts of the retrospective study; n represents number of samples rather than number of patients; percentages might not sum to 100% due to rounding. (C) Examples of Cytosponge analysis on the haematoxylin and eosin stain showing a normal glandular epithelium with intestinal metaplasia and atypia (classed as glandular dysplasia) in a patient with high-grade dysplasia. (D) Immunostaining for TP53 protein on Cytosponge showing normal expression and aberrant overexpression in a Cytosponge sample.

The prevalence of high-grade dysplasia or cancer determined by endoscopic biopsy (primary outcome) was 17% (92 of 557 patients) in the training cohort and 10% (35 of 344) in the validation cohort (figure 1). Patients with high-grade dysplasia or intramucosal cancer in the training cohort were older (Mann-Whitney p=0·013) and had longer Barrett's oesophagus segments (Mann-Whitney p<0·0001) than those with non-dysplastic Barrett's oesophagus or low-grade dysplasia (appendix p 8).

**Figure 2:**
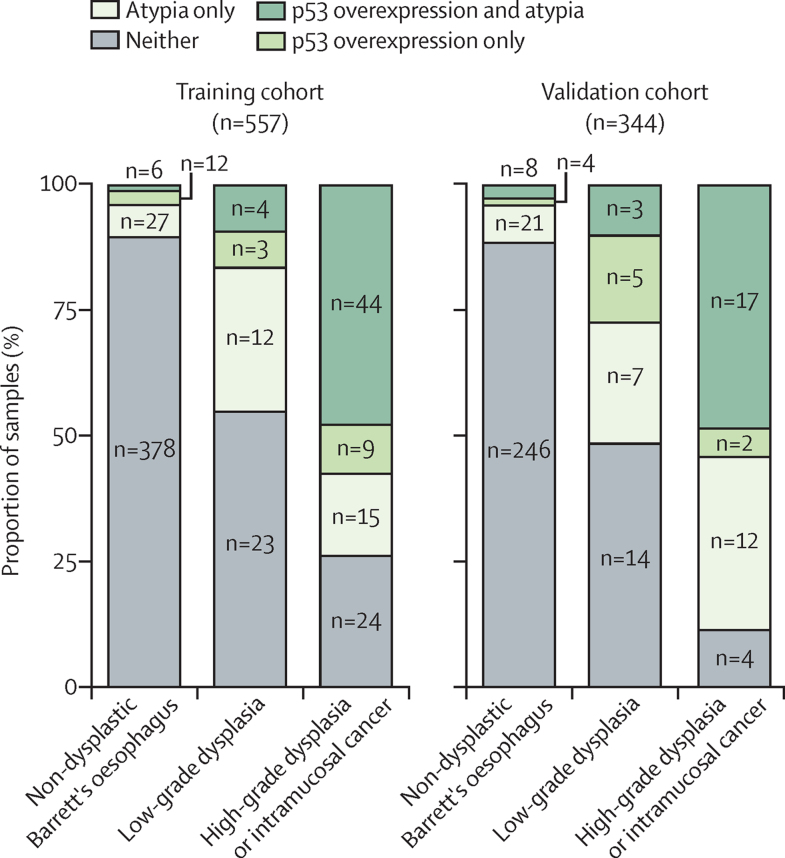
Cytosponge biomarker status compared with gold-standard endoscopic biopsy histopathology diagnosis The distribution of the biomarkers detected on the Cytosponge in individuals diagnosed with non-dysplastic Barrett's oesophagus, low-grade dysplasia, and high-grade dysplasia or intramucosal cancer in the retrospective training and validation cohorts; n represents number of samples. Pathology results missing for one patient in validation cohort.

When considering the Cytosponge result alone, 132 (24%) of 557 patient samples in the training cohort and 80 (23%) of 344 in the validation cohort had atypia, p53 overexpression, or both (figure 2), which resulted in an AUROC for high-grade dysplasia or intramuscosal cancer of 0·80 (95% CI 0·75–0·85) in the training cohort and 0·86 (0·81–0·92) in the validation cohort (table 2, appendix p 9). The sensitivity of the Cytosponge alone was 0·74 (95% CI 0·65–0·83) in the training cohort and 0·89 (0·77–0·97) in the validation cohort; the specificity was 0·86 (0·83–0·89) and 0·84 (0·80–0·88), respectively. When considering any grade of dysplasia (low-grade dysplasia, high-grade dysplasia, or intramucosal cancer), the AUROC was 0·77 (95% CI 0·73–0·81) in the training cohort and 0·80 (0·74–0·86) in the validation cohort (table 2, appendix p 10).

**Table 2:** Diagnostic performance for the three diagnostic models

	**Primary outcome (high-grade dysplasia or cancer)**		**Secondary outcome (any grade of dysplasia or cancer)**	
	Training cohort	Validation cohort	Training cohort	Validation cohort
**Cytosponge biomarker-positive only**				
AUROC	0·80 (0·75–0·85)	0·86 (0·81–0·92)	0·77 (0·73–0·81)	0·80 (0·74–0·86)
Sensitivity	0·74 (0·65–0·83)	0·89 (0·77–0·97)	0·65 (0·57–0·72)	0·72 (0·61–0·83)
Specificity	0·86 (0·83–0·89)	0·84 (0·80–0·88)	0·89 (0·87–0·92)	0·88 (0·84–0·91)
**Cytosponge biomarker-positive plus clinical risk factors**				
AUROC	0·87 (0·82–0·92)	0·91 (0·86–0·95)	0·83 (0·79–0·88)	0·84 (0·78–0·90)
Sensitivity	0·77 (0·68–0·86)	0·80 (0·66–0·91)	0·70 (0·63–0·78)	0·69 (0·56–0·80)
Specificity	0·86 (0·82–0·89)	0·87 (0·83–0·91)	0·86 (0·82–0·89)	0·91 (0·88–0·94)
**Clinical risk factors only**				
AUROC	0·69 (0·63–0·75)	0·72 (0·64–0·80)	0·67 (0·62–0·72)	0·68 (0·61–0·75)
Sensitivity	0·66 (0·57–0·76)	0·91 (0·80–1·00)	0·62 (0·53–0·69)	0·80 (0·69–0·89)
Specificity	0·65 (0·60–0·69)	0·46 (0·40–0·51)	0·65 (0·61–0·70)	0·50 (0·44–0·56)

Data are point estimate (95% CI). The three diagnostic models were Cytosponge biomarker-positive only (atypia or p53 overexpression), Cytosponge biomarker-positive plus a clinical risk factor (age, Barrett's oesophagus segment length, or sex), and clinical risk factors only, which were compared for the primary and secondary outcomes in the retrospective training and validation cohorts, in terms of AUROC, sensitivity, and specificity for each endpoint. For both the primary and secondary endpoints, the Cytosponge biomarker-positive plus clinical risk factor model did not lead to a consistent improvement in performance over the Cytosponge biomarker-positive only model. AUROC=area under the receiver operating characteristic curve.

Only two clinical demographic variables were identified as significantly associated with primary or secondary outcome in a multivariate analysis, which were Barrett's oesophagus segment length and patient age (sex also had borderline significance; appendix pp 6–7). However, clinical variables alone were a poor predictor of primary or secondary outcome (table 2).

Combining the clinical risk factors of Barrett's oesophagus segment length, patient age, and sex with the Cytosponge biomarkers to generate probabilities in a new logistic regression model did not improve performance compared with the Cytosponge biomarkers alone. In the training cohort, the sensitivity of Cytosponge biomarkers combined with clinical risk factors was 0·77 (95% CI 0·68–0·86). In the validation cohort, the sensitivity of the combination was 0·80 (0·66–0·91), which was marginally worse than for the Cytosponge biomarkers alone (table 2, appendix p 10), although AUROC and specificity were higher.

Hence, cellular atypia and p53 overexpression on Cytosponge, with or without clinical risk factors, defined the high-risk group. The risk for the primary outcome (high-grade dysplasia or cancer) in the biomarker-positive group was 52% (68 of 132 patients) in the training cohort and 41% (31 of 75) in the validation cohort. The Cytosponge biomarkers results were therefore used to define the first decision point on the decision tree.

The second point on the decision tree used the clinical demographic risk factors (Barrett's oesophagus segment length, age, and sex) to identify individuals with dysplasia. 19 (79%) of 24 patients in the training cohort and two (50%) of four patients in the validation cohort, with high-grade dysplasia or intramucosal cancer, who were negative for Cytosponge biomarkers, had a clinical demographic risk factor and were part of this moderate-risk group (appendix p 5).

**Figure 3:**
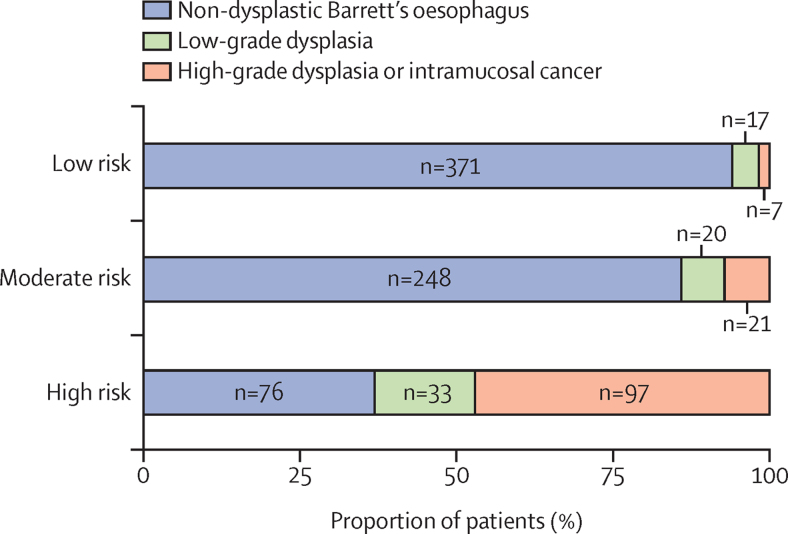
Summary of dysplasia status in each risk group The combined retrospective training and validation cohorts (total 891 patients, one in the validation cohort was missing pathology information) were scored using the decision tree and patients were classified as high risk (biomarker-positive), moderate risk (clinical risk factor; Barrett's oesophagus length, sex, or age), and low risk (neither clinical risk factor nor pathology biomarkers were present; appendix p 5). The high-risk group shows a high proportion of patients who were diagnosed endoscopically with high-grade dysplasia or intramucosal cancer. The moderate risk group includes most of the remaining patients who were diagnosed with high-grade dysplasia or intramucosal cancer and low-grade dysplasia, which suggests that this group would benefit from a more vigilant Cytosponge surveillance strategy or Cytosponge alternated with endoscopy. The high-risk and moderate-risk groups combined had an overall sensitivity for high-grade dysplasia or cancer in the combined retrospective training and validation cohorts of 0·94 (95% CI 0·91–0·98).

When evaluating the high-risk (Cytosponge biomarkers) and moderate-risk (clinical risk factors) groups versus the low-risk group (no biomarker or clinical risk factors), there was an overall sensitivity in the combined retrospective training and validation cohorts of 0·94 (95% CI 0·91–0·98) for high-grade dysplasia or intramucosal cancer and 0·88 (0·83–0·92) for any grade of dysplasia in the combined risk group. The risk of high-grade dysplasia or cancer in the low-risk group was 2% (seven of 395 patients) in the combined retrospective cohorts (2% [five of 210] in the training cohort and 1% [two of 185] in the validation cohort; figure 3). Hence, the likelihood of diagnosing high-grade dysplasia or cancer at endoscopy for Cytosponge biomarker-positive patients was three times higher than the current clinical practice of endoscopic surveillance for all (47% [97 of 206 patients] *vs* 14% [125 of 891]).

In the retrospective study, six patients who did not have dysplasia detected at their confirmatory endoscopy subsequently had dysplasia detected at follow-up endoscopy, five of whom had endoscopic therapy; three patients had progressed to high-grade dysplasia or intramucosal cancer, two had a low-grade dysplasia diagnosis confirmed on two occasions, meeting the guidelines for endoscopic treatment, and one patient had low-grade dysplasia on one occasion that was then downgraded to non-dysplastic Barrett's oesophagus.

**Figure 4:**
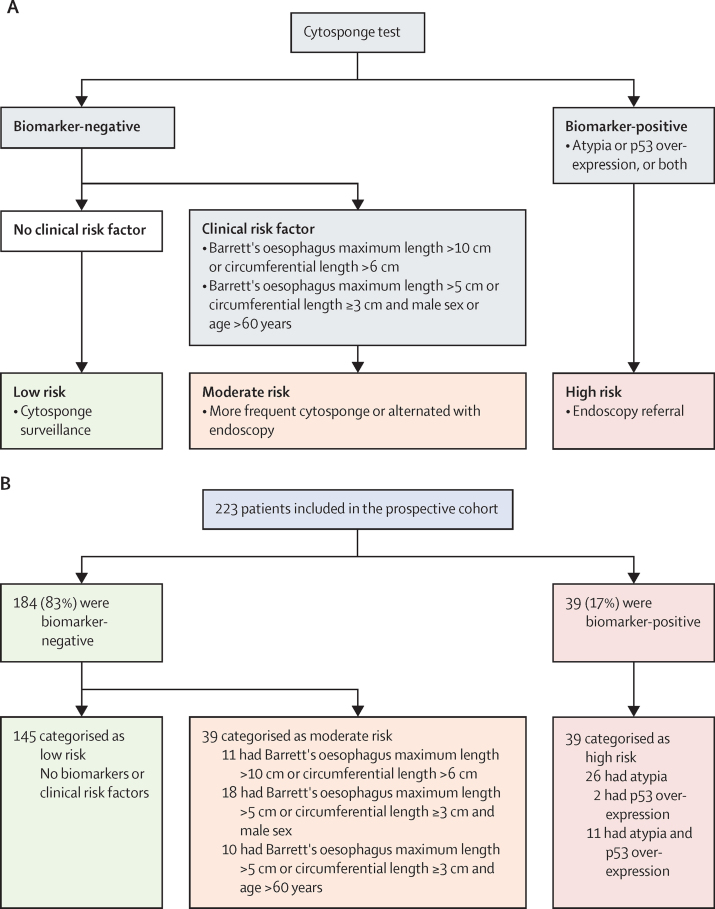
Decision tree in real-world prospective pilot (A) The Cytosponge decision tree for high-risk (biomarker-positive), moderate-risk (biomarker-negative with clinical risk factor), and low-risk (biomarker-negative and no clinical risk factor) groups; each risk group is presented with possible clinical decisions for endoscopy timing. (B) The prospective cohort findings following the decision tree based on the Cytosponge pathology result and clinical risk factors; 44 patients did not have segment length information, of whom 32 were Cytosponge biomarker-negative and considered low risk.

223 patients were prospectively assessed during the COVID-19 pandemic (table 1). The Cytosponge results were returned within a median of 8 days (IQR 6–11); 39 (17%) of 223 patients were biomarker positive and recommended for endoscopy: 26 (12%) of 223 had cellular atypia only, two (1%) of 220 had p53 overexpression only, and 11 (5%) of 223 had both (figure 4). When compared with endoscopic biopsy results, this yielded a positive predictive value of 31% (12 of 39 patients) for high-grade dysplasia or intramucosal cancer and 44% (17 of 39) for any grade of dysplasia; as expected, patients with both aberrant p53 expression and atypia were most likely to have high-grade dysplasia or cancer (seven [64%] of 11 patients). Applying the clinical risk factors (Barrett's oesophagus length, age, and sex) identified 39 (17%) of 223 patients as having moderate risk. The low-risk group comprised 145 (65%) of 223 patients (figure 4B), including 32 patients in whom Prague (M and C) lengths were not recorded making the low versus moderate evaluation incomplete. The appendix (p 11) illustrates how the decision tree can be utilised in the clinical setting.

## Discussion

We present a new triage approach for Barrett's oesophagus surveillance in which the patient pathway is tailored according to their risk of high-grade dysplasia or early cancer. The Cytosponge p53 immunohistochemistry and atypia result identifies individuals at high risk of dysplasia or cancer, with a three times greater enrichment compared with the current practice of performing endoscopy for all patients in surveillance. Furthermore, a low-risk group (patients who are Cytosponge biomarker-negative and have no clinical risk factors) was identified who could have less burdensome follow-up.

The decision tree presented here is compatible with clinical decision making. Reliance on Cytosponge results alone as the first point in the decision tree has the advantage that the clinician can make a management decision directly from the pathology report. In our Cytosponge-based decision tree, the report would enable the high-risk patients to be easily identified and triaged to endoscopy. Patients without positive biomarkers but with known clinical risk factors are categorised into the moderate-risk group. If another Cytosponge (or endoscopy) is performed within, for example, 12 months for this group, it should provide a good safety net to minimise the risk of missing dysplasia. Patients with a negative Cytosponge and no clinical risk factors would continue surveillance with Cytosponge according to the recommended intervals from the societal guidelines. One could thus envisage a redesigned clinical surveillance pathway in which alternating Cytosponge and endoscopy could be used for patients at moderate risk with longer non-dysplastic Barrett's oesophagus segments, whereas those at lowest risk could continue Cytosponge surveillance until such time when their biomarker status changes. Based on our data, this redesigned surveillance pathway could substantially reduce the endoscopy burden. Further validation and health economic modelling will be helpful to inform how frequently Cytosponge should be repeated and model different options.

For the evaluation of new diagnostic tests, comparison with the current clinical standard is required. For the retrospective cross-sectional study, we used endoscopy as the gold standard but we acknowledge that this is also not the perfect test, as endoscopic punch biopsies are subject to sampling bias. Post-endoscopy incident oesophageal cancer is estimated to account for 14% of the oesophageal cancer burden^21^ and missed dysplasia at index endoscopy accounts for around 10% of cancer cases.^22^, ^23^ In a randomised crossover trial with two endoscopies performed 6 weeks apart (in 153 patients) to assess the utility of confocal endomicroscopy, inconspicuous dysplasia was missed by the standard white-light Seattle protocol in approximately 50% of cases.^24^ Low-grade dysplasia is, however, prone to overdiagnosis unless diagnosed by consensus.^20^, ^25^ It is therefore pertinent to consider those cases with atypia and p53 overexpression on Cytosponge who did not have dysplasia detected at their confirmatory endoscopy (false positives). Follow-up data are incomplete for the prospective cohort, but in the retrospective study, six such patients had dysplasia detected at subsequent follow-up endoscopy. Hence, neither endoscopy nor Cytosponge is the perfect test and dysplasia can be missed; a combined approach using Cytosponge as a triage test might improve the yield of Barrett's oesophagus surveillance.

It is also important to note that because assessment of the Cytosponge biomarkers relies on review by a pathologist, it might be subject to observer bias and variability. In the future, machine learning might help to homogenise pathology scoring and reduce pathologist assessment time, as we have previously shown for *TFF3*.^26^ We have also shown that a relatively simple measurement of the genomic alterations in a biopsy can predict future risk of cancer up to 8 years in advance.^27^ Application of this method to Cytosponge samples might further improve the sensitivity of the model for detecting prevalent low-grade dysplasia and high-grade dysplasia and might also predict future cancer risk.

This study has several strengths, such as the large number of patients. The decision tree uses simple, objective, and reproducible variables and the laboratory reporting of Cytosponge was performed to a clinical standard. It should be noted that as processing and pathology for the Cytosponge are not standard provisions in a pathology laboratory, a centralised service was provided to ensure quality control of the samples. As discussed, no diagnostic gold standard is perfect; however, the endoscopists who participated in this study are clinicians running an established Barrett's oesophagus surveillance service and endoscopic images were reviewed for quality assurance in the BEST2 and BEST3 cohorts. Another strength is the inclusion of multiple community hospitals, where the majority of surveillance takes place.

This study also has some limitations. The original study definitions for the published BEST2 and BEST3 cohorts prioritised longer Barrett's oesophagus segments, which could limit the sensitivity of the Cytosponge biomarkers in shorter segments. However, in this study, we included all available patients. Although the retrospective training cohort had a greater proportion of patients with a Barrett's oesophagus length of greater than 3 cm, 3 cm was the median length in the validation cohort and there was no exclusion by segment length in the prospective cohort. In the training cohort from BEST2, the prevalence of high-grade dysplasia or intramuscosal cancer was 17% (92 of 557 patients); however, the validation cohort was more representative of the target population, with a prevalence of 10% (33 of 334). For the prospective study, the follow-up data for those not prioritised for endoscopy are awaited and are likely to be delayed during the COVID-19 pandemic, but the prevalence of high-grade dysplasia or intramucosal cancer at the cutoff date for this analysis was 5% (12 of 223 patients). In the future, a further prospective evaluation of this decision tree coupled with endoscopy follow-up information will be informative. As risk calculators and machine learning approaches become more commonplace in clinical practice, this decision tree approach might be refined further.

In conclusion, we present a Cytosponge pathology test coupled with a decision tree algorithm that estimates the likelihood of detecting Barrett's oesophagus-related neoplasia in patients undergoing surveillance. Further evaluation of this approach is warranted to realise the potential of a method which could relieve the surveillance burden on patients and clinicians, allowing endoscopy to be reserved for those requiring specialist assessment and intervention for dysplasia.

**This online publication has been corrected. The corrected version first appeared at thelancet.com/oncology on January 31, 2022**

## Data sharing

Individual patient-level data are available for the retrospective cohorts in the BEST2 and BEST3 trials upon request. Patient-level data included in the DELTA prospective trial will be available on completion of the trial in April, 2023. Trial protocols are available via the University of Cambridge data repository at https://www.data.cam.ac.uk/repository.

## Declaration of interests

The Cytosponge technology including the device and TFF3 biomarker has been licensed by the Medical Research Council to Covidien (now Medtronic). RCF and MO'D are named on patents related to this test. RCF and MO'D are shareholders for Cyted. MO'D is a consultant for Cyted. All other authors declare no competing interests.
