# Correction to *Lancet Oncol* 2022; 23: 270–78

**DOI:** 10.1016/S1470-2045(22)00026-2

**Published:** 2022-02

*Pilonis ND, Killcoyne S, Tan WK, et al. Use of a Cytosponge biomarker panel to prioritise endoscopic Barrett's oesophagus surveillance: a cross-sectional study followed by a real-world prospective pilot.* Lancet Oncol *2022; **23:** 282–90*—In this Article, figure 2 was incorrect and has been updated. This correction has been made to the online version as of Jan 31, 2022, and the printed version is correct.

