# Supplementary material for: Use of a Cytosponge biomarker panel to prioritise endoscopic Barrett's oesophagus surveillance: a cross-sectional study followed by a real-world prospective pilot
Source: Lancet Oncol. 2022 Feb;23(2):270–8. doi: 10.1016/S1470-2045(21)00667-7 (PMC8803607; doi:10.1016/S1470-2045(21)00667-7)
Supplement: Supplementary appendix [file mmc1.pdf]

THE LANCET
Oncology

Supplementary appendix

This appendix formed part of the original submission and has been peer reviewed. We post it as supplied by the authors.

Supplement to: Pilonis ND, Killcoyne S, Tan WK, et al. Use of a Cytosponge biomarker panel to prioritise endoscopic Barrett's oesophagus surveillance: a cross-sectional study followed by a real-world prospective pilot. *Lancet Oncol* 2022; published online Jan 11. https://doi.org/10.1016/S1470-2045(21)00667-7.

Supplemental Methods

Study procedures and definitions

Cytosponge specimens were processed centrally by the Addenbrooke's Hospital Human Research Tissue Bank, as previously described for patients from the BEST2 and BEST3 cohorts(1) and by the Cyted Ltd laboratory, Huntingdon, UK for patients included as part of the DELTA study. To qualify for inclusion in the study all patients had endoscopic evidence of columnar epithelium in the distal oesophagus with a minimum length of 1 cm (tongues or circumferential using the Prague C, M criteria) containing intestinal metaplasia on histopathological examination and TFF3 positive cells confirming intestinal metaplasia on Cytosponge as previously described.(2,3) Formalin fixed and paraffin-embedded Cytosponge samples were cut into consecutive sections. To ensure consistency, the first slide containing two sections was stained with haematoxylin and eosin (H&E) to identify for the presence of glandular atypia and the fourth slide was used for p53 IHC. A positive biomarker test is regarded as the presence of atypia and/or aberrant p53 IHC as assessed by at least two out of four expert pathologists (M.O., S.M., A.M., M.T.). Glandular atypia included clear cut dysplasia and those graded as 'atypia of unknown significance'. A p53 staining with an intensity of 3 was considered significant, as previously published(4). Although the absence of p53 staining also constitutes abnormal expression in a small proportion of patients with a *TP53* mutation, this cannot be reliably ascertained due to a lack of wild type basal cell staining on Cytosponge samples.(5) Consensus agreement for p53 over-expression and atypia between pathologists was used in any case of uncertainty.

The endoscopies were carried out by local study endoscopists and biopsies were performed using the recommended Seattle biopsy protocol whereby 4-quadrant biopsies were taken every 2cm of BO length and targeted biopsies taken for any visible nodular lesion. (6) Diagnostic biopsies were reviewed locally, and any dysplasia diagnoses were reviewed by in a central 1

pathology consensus meeting with four expert pathologists who were blinded to the Cytosponge tests. Results of the highest histopathological diagnosis of dysplasia from endoscopic biopsies taken following the Cytosponge procedure were used as a gold-standard reference. 91% (509/557) and 49% (165/344) of the training and validation cohorts had endoscopy on the same day as the Cytosponge, 7% (38/557) and 35% (122/344) were a median of 1 week between procedures. There was missing information on endoscopy dates for 12 patients in the training set and 57 patients in the validation cohort.

Due to restrictions in performing aerosol generating endoscopy procedures during the Covid 19 pandemic (from March 2020), patients with NDBO undergoing BO surveillance were offered the Cytosponge biomarker panel test as alternative to endoscopy as part of the DELTA study. Results were processed in a clinically approved central laboratory (Cyted Ltd). Patients with a positive biomarker (atypia and/or p53 IHC) result from the Cytosponge were referred for endoscopy within 3 months. Surveillance endoscopies for patients who tested negative on the Cytosponge were postponed with a plan to perform endoscopy when available and ideally within the maximum interval recommended BSG guidelines; however for most patients these follow-up endoscopy procedures are not yet available and these data are not required for the purposes of this study.

Decision Tree

Decision tree clinical parameters for the 'moderate' risk group were developed based on the published risks for long-segment BO, patient age, and sex(7,8). We performed additional analyses on our retrospective training cohort to identify cutoffs that maximised sensitivity for the primary and secondary endpoints. Of the 24 patients diagnosed with HGD/IMC that were negative for both atypia and p53, 7 had BO segments that were in the highest quantile for

Prague lengths (M>10cm or C>6), 12 patients had BO segments longer than the median (5cm) and were either male or over 60 years at the time of the test. These clinical variables captured 79% (19/24) of the biomarker negative patients with HGD/IMC. The remaining 5 patients were short segment BO (≤3cm) and while this could indicate sensitivity limitations with respect to short segments, 15 of the 132 biomarker-positive 'high' risk cases were ≤3cm and were not subsequently diagnosed with HGD/IMC. Suggesting that false positives may be more likely than false negatives in these cases.

References

1. Ross-Innes CS, Debiram-Beecham I, O'Donovan M, Walker E, Varghese S, Lao-Sirieix P, et al. Evaluation of a Minimally Invasive Cell Sampling Device Coupled with Assessment of Trefoil Factor 3 Expression for Diagnosing Barrett's Esophagus: A Multi-Center Case–Control Study. Franco EL, editor. PLOS Med [Internet]. 2015 Jan 29 [cited 2015 Oct 19];12(1):e1001780. Available from: http://journals.plos.org/plosmedicine/article?id=10.1371/journal.pmed.1001780
2. Fitzgerald RC, di Pietro M, O'Donovan M, Maroni R, Muldrew B, Debiram-Beecham I, et al. Cytosponge-trefoil factor 3 versus usual care to identify Barrett's oesophagus in a primary care setting: a multicentre, pragmatic, randomised controlled trial. Lancet [Internet]. 2020 Aug 1 [cited 2020 Dec 15];396(10247):333–44. Available from: www.thelancet.com
3. Gehrung M, Crispin-Ortuzar M, Berman AG, O'Donovan M, Fitzgerald RC, Markowetz F. Triage-driven diagnosis of Barrett's esophagus for early detection of esophageal adenocarcinoma using deep learning. Nat Med [Internet]. 2021 May 1 [cited 2021 Jun 10];27(5):833–41. Available from: https://doi.org/10.1038/s41591-021-01287-9
4. Kaye P V. p53 Immunohistochemistry as a biomarker of dysplasia and neoplastic progression in Barrett's oesophagus. Diagnostic Histopathol. 2015 Mar 1;21(3):89–98.
5. Ross-Innes CS, Chettouh H, Achilleos A, Galeano-Dalmau N, Debiram-Beecham I, MacRae S, et al. Risk stratification of Barrett's oesophagus using a non-endoscopic sampling method coupled with a biomarker panel: a cohort study. Lancet Gastroenterol Hepatol [Internet]. 2017 Jan [cited 2017 Feb 10];2(1):23–31. Available from: http://linkinghub.elsevier.com/retrieve/pii/S2468125316301182
6. Levine DS, Blount PL, Rudolph RE, Reid BJ. Safety of a systematic endoscopic biopsy protocol in patients with Barrett's esophagus. Am J Gastroenterol. 2000 May 1;95(5):1152–7.
7. Hamade N, Vennelaganti S, Parasa S, Vennalaganti P, Gaddam S, Spaander MCW, et al. Lower Annual Rate of Progression of Short-Segment vs Long-Segment Barrett's Esophagus to Esophageal Adenocarcinoma. Clin Gastroenterol Hepatol. 2019 Apr 1;17(5):864–8.
8. Parasa S, Vennalaganti S, Gaddam S, Vennalaganti P, Young P, Gupta N, et al. Development and Validation of a Model to Determine Risk of Progression of Barrett's Esophagus to Neoplasia. Gastroenterology [Internet]. 2018 Apr 1 [cited 2020 Feb

12];154(5):1282-1289.e2. Available from: http://www.ncbi.nlm.nih.gov/pubmed/29273452

Ethical Approval

Ethical approval for the BEST2 study was obtained from the East of England–Cambridge Central Research Ethics Committee (No: 10/H0308/71) also registered in the UK Clinical Research Network Study Portfolio (9461). The BEST3 trial was registered with the ISRCTN registry, number ISRCTN68382401 and ISRCTN 91655550 for DELTA. Written informed consent was obtained from each participant.

Patient and Public Involvement

Members of the Registered Charities Heartburn Cancer UK and Action Against Heartburn have assisted with devising patient-facing materials for Cytosponge and are members of the DELTA trial steering committee.

Supplemental Tables and Figures

Supplemental Table 1. Significant coefficients associated with the primary (HGD/IMC) and secondary (any dysplasia) endpoints. Of the clinical features examined only BO length in cm (M) and patient age in years were associated and considered in subsequent risk prediction. Atypia and p53 show a much larger effect as shown by the odds ratios in both models.

	**Primary endpoint: HGD/IMC**		**Secondary endpoint: Any dysplasia**	
**Coefficient**	**Odds ratio**	**95% CI**	**Odds ratio**	**95% CI**
p53 over expression	12.1	6.25 - 23.69	8.3	4.33 – 16.26
Atypia	5.23	2.81 – 9.70	6.6	3.78 - 11.6
M (cm)	1.15	1.09 - 1.23	1.13	1.08 - 1.20
Age (years)	1.02	1.00 – 1.05	1.03	1.01 - 1.05

Supplemental Table 2: Patient counts by pathology for the risk categories for the retrospective training and validation cohorts. These numbers are reflected by Figure 3 in the main text. The sensitivity of the high and moderate risk groups for HGD/IMC is 0.94 for the combined training and validation cohorts.

	**HGD/IMC**		**LGD**		**NDBO**	
**Risk classes**	**Training (n=92)**	**Validation (n=33)**	**Training (n=42)**	**Validation (n=28)**	**Training (n=423)**	**Validation (n=272)**
**High (biomarker+)**	68/92	29/33	19/42	14/28	45/423	31/272
**Moderate (clinical factors)**	19/92	2/33	14/42	6/28	182/423	66/272
**Low**	5/92	2/33	9/42	8/28	196/423	175/272

Supplemental Table 3: Univariate logistic regression analysis for each clinical variable for the primary outcome (HGD/IMC). Significant variables are then tested in a multivariate analysis shown in Supplemental Table 1.

**Variable**	**p-value**	**estimate**
**p53IHC**	4.44E-27	3.17E+00
**GlandularAtypia**	9.08E-25	2.72E+00
**M**	1.53E-06	1.49E-01
**C**	4.69E-04	1.00E-01
**Age**	4.41E-03	3.83E-02
**EverSmoked**	3.87E-02	5.17E-01
**Gender**	7.40E-02	6.10E-01
**WaistHipRatio**	8.48E-02	2.13E+00
**Oesophagitis**	8.60E-02	-1.26E+00
**CurrentAlcoholFreq**	1.25E-01	5.23E-01
**Family_history_EAC**	1.93E-01	-7.00E-01
**YearsHeartburn**	3.19E-01	2.89E-01
**BMI**	3.84E-01	2.02E-02
**Ethnicity**	5.10E-01	6.95E-01
**Cyto_to_Endo_Wks**	5.59E-01	-1.03E-01
**TFF3 positive count**	6.33E-01	-2.18E-04
**HiatusHernia**	7.98E-01	7.69E-02
**Centre**	9.50E-01	5.01E-02
**CurrentAlcoholUnitsPerWeek**	9.54E-01	-4.25E-04

Supplemental Table 4: Univariate logistic regression analysis for each clinical variable for the secondary outcome (LGD/HGD/IMC). Significant variables are then tested in a multivariate analysis shown in Supplemental Table 1.

Variable	p-value	estimate
**GlandularAtypia**	4.17E-27	2.71E+00
**p53IHC**	1.41E-22	2.90E+00
**M**	1.72E-06	1.36E-01
**Years_DiagnosisToCytosponge**	1.42E-04	-9.30E-02
**Age**	6.74E-04	3.76E-02
**C**	8.40E-04	8.64E-02
**Gender**	6.11E-03	8.40E-01
**EverSmoked**	2.86E-02	4.67E-01
**Oesophagitis**	3.11E-02	-1.32E+00
**WaistHipRatio**	3.43E-02	2.51E+00
**CurrentAlcoholFreq**	8.50E-02	5.07E-01
**HiatusHernia**	1.39E-01	4.08E-01
**YearsHeartburn**	1.49E-01	3.64E-01
**TFF3 positive count**	2.61E-01	-5.17E-04
**Family_history_EAC**	3.86E-01	-3.51E-01
**CurrentAlcoholUnitsPerWeek**	4.55E-01	-5.25E-03
**Cyto_to_Endo_Wks**	5.92E-01	-7.03E-02
**BMI**	6.45E-01	9.62E-03
**Ethnicity**	6.45E-01	3.63E-01
**Centre**	8.78E-01	-1.05E-01

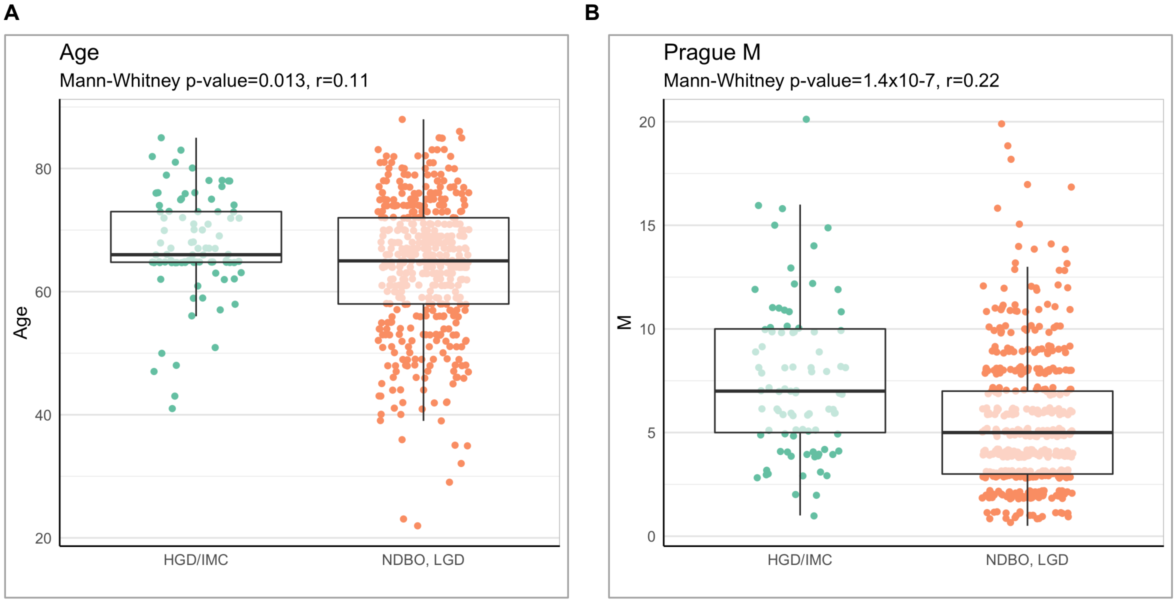

Supplemental Figure 1: Endoscopic diagnosis of HGD/IMC versus NDBO and LGD show a significant difference (Mann-Whitney test) for patient age and BO segment length.

A

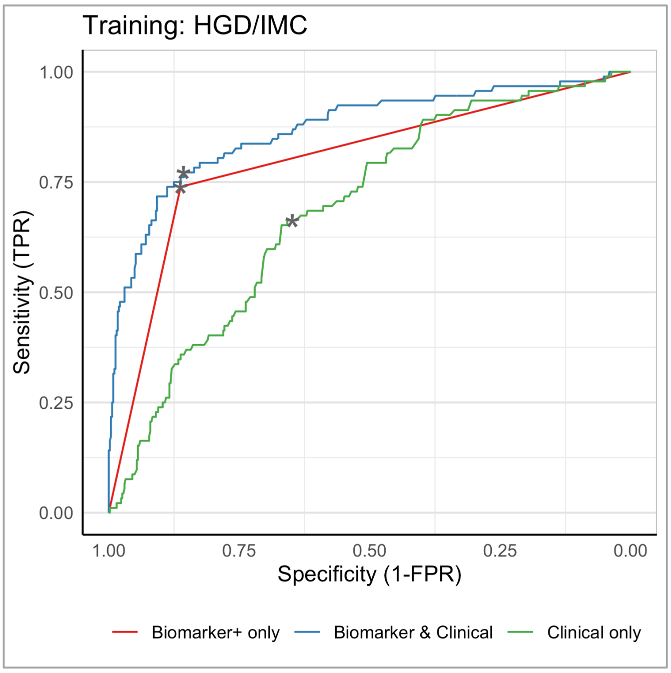

B

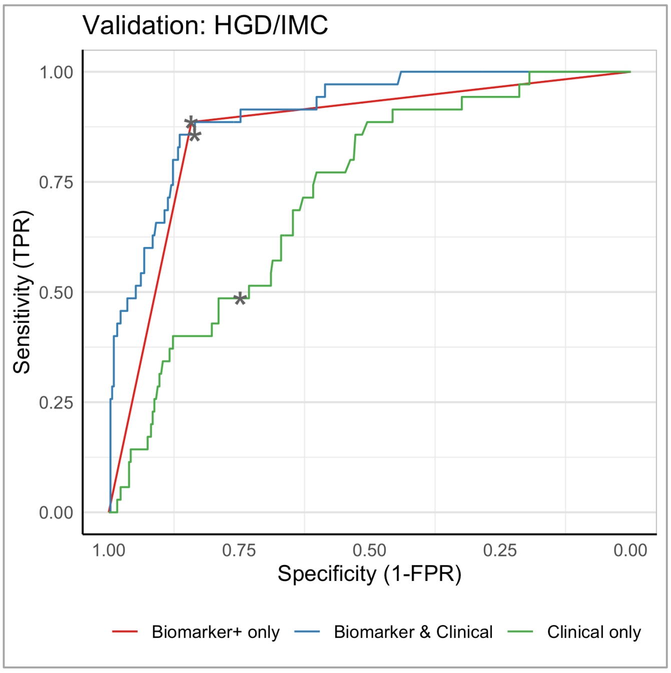

Supplemental Figure 2: ROC AUC plots corresponding to Table 1 for three diagnostic models: biomarker-positive(+) only (atypia or p53), biomarker+ with age and BO length (M), compared to clinical factors alone (age and length) for the primary (HGD/IMC) outcome. The asterisk in each shows the best fit threshold (balancing the sensitivity and specificity) for each curve. An important note for the biomarker-positive(+) only curve is that this is based on a binary predictor (biomarker is positive or negative) resulting in only a single threshold and so is shown only to provide a comparison to the multi-predictor models.

A

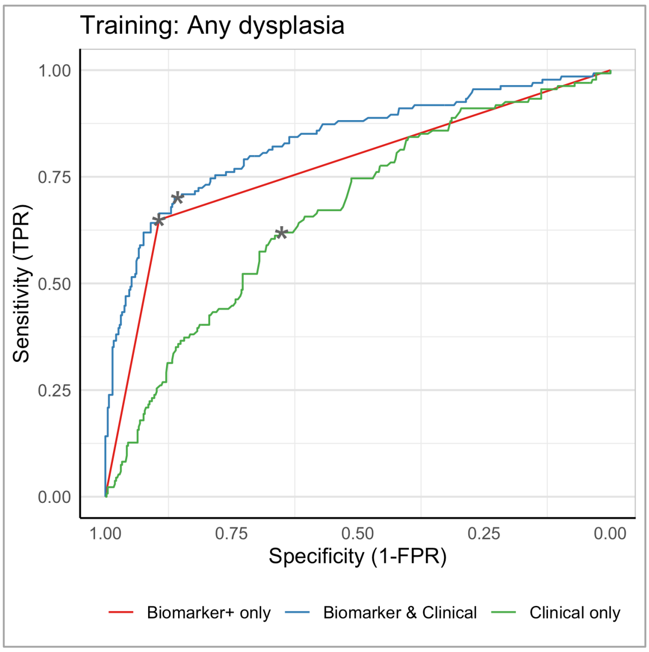

B

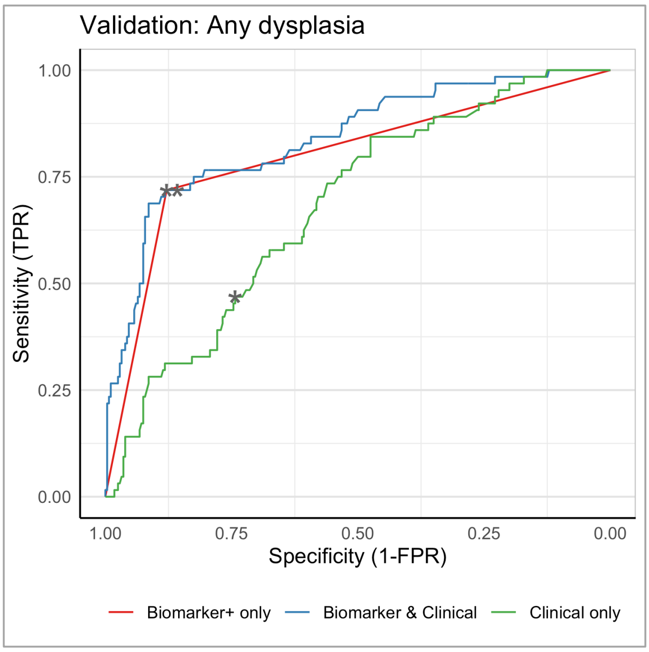

Supplemental Figure 3: ROC AUC plots corresponding to Table 1 for three diagnostic models: biomarker-positive(+) only (atypia or p53), biomarker+ with age and BO length (M), compared to clinical factors alone (age and length) for the secondary (any dysplasia) outcome. The asterisk in each shows the best fit threshold (balancing the sensitivity and specificity) for each curve. An important note for the biomarker-positive(+) only curve is that this is based on a binary predictor (biomarker is positive or negative) resulting in only a single threshold and so is shown only to provide a comparison to the multi-predictor models.

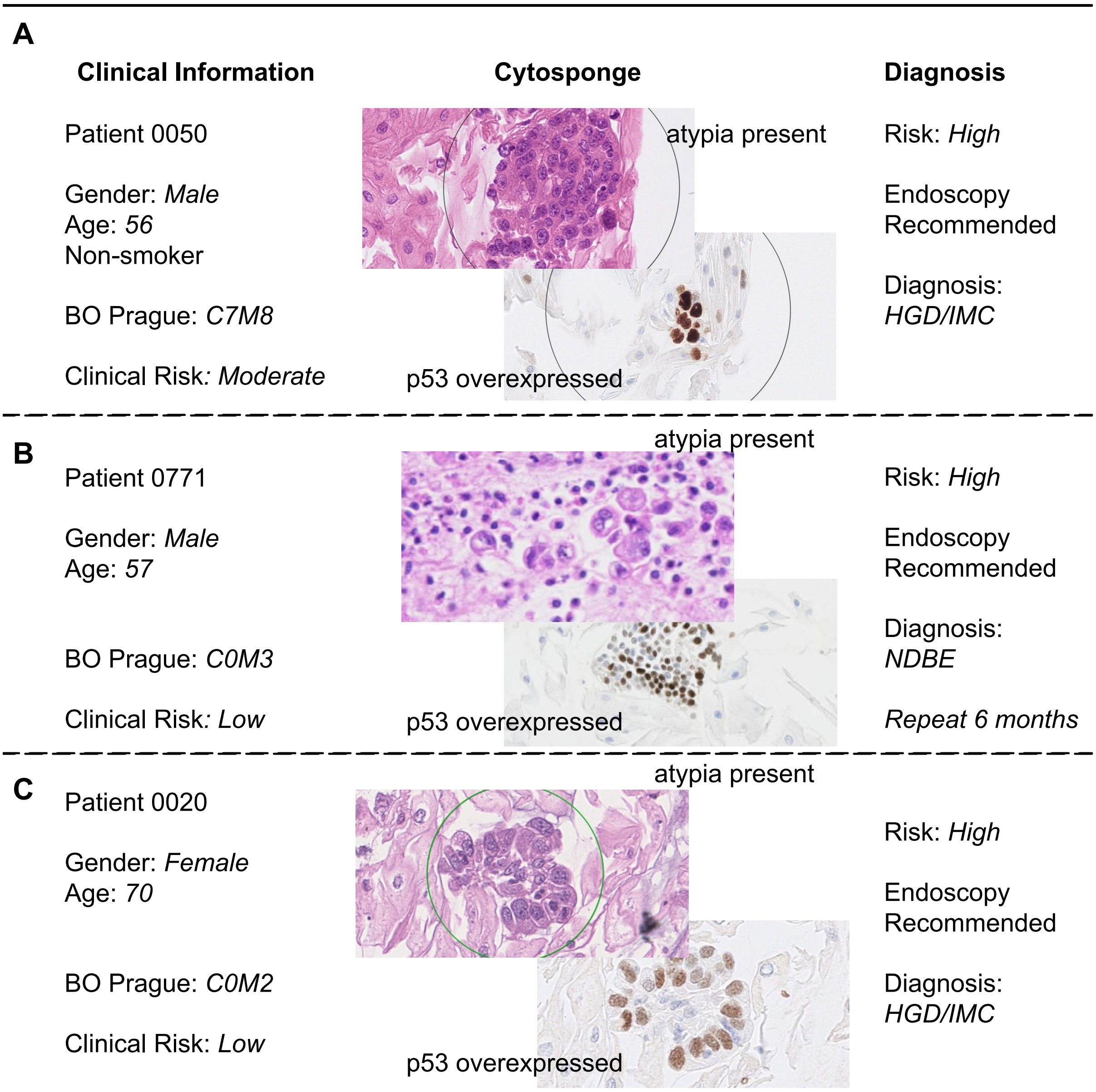

Supplemental Figure 4: The utility of the decision tree is shown in these case studies from the prospective cohort. (**A**) 56 year old male patient, with a C9M10 BO segment had a Cytosponge showing overexpressed p53 and atypia putting them in the high risk category. An urgent endoscopy did not show any suspicious endoscopic features. Biopsies taken in accordance with the Seattle protocol, showed HGD and IMC.(**B**) 57 year old male patient, with a C0M3 BO segment was positive for both biomarkers but the endoscopic biopsies did not show any abnormality. The clinical characteristics mean that this patient would not fulfil the moderate risk criteria, but the Cytosponge atypia and p53 abnormalities are concerning (Figure 4C, middle panel). He will be re-scoped in 6 months on the basis that dysplasia may have been missed due to sampling bias at endoscopy. (**C**) A 70 year old female patient, with a C0M2 segment, also considered clinically low risk, was found to have atypia and p53 on Cytosponge with a nodule containing IMC confirmed at endoscopy.
